# Tauroursodeoxycholic acid-induced increase in ectopic muscle mineralization occurs exclusively in dystrophic muscles and is independent of endoplasmic reticulum stress

**DOI:** 10.1038/s41598-025-21534-0

**Published:** 2025-10-28

**Authors:** Justyna Róg, Dorota Dymkowska, Bernadeta Michalska, Olga Krupska, Krzysztof Milewski, Paweł Matryba, Artur Wolny, Monika Pawłowska, Łukasz Bożycki, Dawid Stępnik, Dariusz C. Górecki, Krzysztof Zabłocki

**Affiliations:** 1 Laboratory of Cellular Metabolism, Nencki Institute of Experimental Biology PAS, 3 Pasteur Street, 02-093 Warsaw, Poland; 2 Department of Immunology, Medical University of Warsaw, 5 Nielubowicz Street, 02-097 Warsaw, Poland; 3 Laboratory of Neurobiology, BRAINCITY, Nencki Institute of Experimental Biology PAS, 3 Pasteur Street, 02-093 Warsaw, Poland; 4 Department of Radiology I, The Maria Sklodowska-Curie National Research Institute of Oncology in Warsaw, Roentgen Street 5, 02-781 Warsaw, Poland; 5 Laboratory of Imaging Tissue Structure and Function, Nencki Institute of Experimental Biology PAS, 3 Pasteur Street, 02-093 Warsaw, Poland; 6 Laboratory of Biochemistry of Lipids, Nencki Institute of Experimental Biology PAS, 3 Pasteur Street, 02-093 Warsaw, Poland; 7 Laboratory of Cytometry, Nencki Institute of Experimental Biology PAS, 3 Pasteur Street, 02-093 Warsaw, Poland; 8 School of Medicine, Pharmacy and Biomedical Sciences, University of Portsmouth, St Michael’s Building, White Swan Road, Portsmouth, PO1 2DT UK

**Keywords:** Biochemistry, Cell biology, Pathogenesis

## Abstract

Calcification of dystrophic skeletal muscles was described previously and attributed, among others, to ER-stress, elevated phosphate concentration and chronic inflammation. Tauroursodeoxycholic acid (TUDCA) is considered an artificial chaperone protecting cells against ER-stress thus could prevent an ectopic mineralisation of soft tissues. Because an enhanced ER-stress is a feature of dystrophic muscles and it promotes soft tissue mineralisation we hypothesised that TUDCA treatment should reduce mineral deposits in dystrophic skeletal muscles, and tested this concept using two mouse models of DMD. Four-week old mdx, mdx^βetageo^ and w/t mice were administered TUDCA in drinking water for 4 weeks. At 8 weeks, following tissue-clearing and calcium minerals staining with alizarin, mineralisation was evaluated using whole body scanning. Additionally, isolated skeletal muscles were analysed by Western blotting for ER-stress and calcification markers, and using various microscopic methods. Enzymatic activity of alkaline phosphatase was also assayed. Unexpectedly, TUDCA enhanced calcification of dystrophic but not dystrophin-positive muscles. TUDCA did not affect the elevated ER-stress markers found in dystrophic muscles nor impact pro-calcifying proteins RUNX2, Osterix and BMP2/4, which were also overexpressed in dystrophic muscles. The alkaline phosphatase levels, which were reduced in dystrophic muscles, were not affected by this treatment. The increase in ectopic calcification in dystrophic muscles induced by TUDCA is specific to muscles lacking dystrophin. This effect is not linked to the alleviation of ER stress or the overexpression of proteins directly involved in calcium mineral accumulation.

**Supplementary Information:**

The online version contains supplementary material available at 10.1038/s41598-025-21534-0.

## Introduction

Duchenne muscular dystrophy (DMD) is a debilitating and incurable X-linked inherited disease^1^. Death is caused by the progressive degeneration of striated muscles aggravated by sterile inflammation^2^. Eventually, DMD patients die from cardiac and respiratory complications.

However, the pleiotropic effects of the mutant gene also include neuropsychiatric^3^ and bone mineral abnormalities^4,5^. These widespread symptoms stem from the complexity of the mutant gene that encodes numerous dystrophin isoforms. Mutations affecting the full-length (427 kDa) dystrophins are necessary and sufficient to trigger the disease. Loss of short isoforms (Dp260, Dp140, Dp116 and Dp71), can exacerbate the dystrophic phenotype, often in a tissue-specific manner^6^. It is now clear that Dp427 role extends beyond linking the myofiber cytoskeleton, the cell membrane and the extracellular matrix^7^ into the normal functioning of satellite cells^8^ and myoblasts^9^. In fact, loss of its expression results in common as well as cell-specific abnormalities not only causing DMD but even impacting malignant tissues^10^.

Interestingly, increased intracellular calcium is a common abnormality found in all dystrophic muscle cells, both developmentally^11^ and in adults, as well as in tissues dissimilar to muscle, provided they exhibit altered expression of the DMD gene^12^.

This calcium dys-homeostasis coincides with ectopic muscle calcification is a pathological feature in both, animal models and patients found as early as in 12-week-old DMD foetuses^13^. The origins of ectopic calcification in soft tissues under various pathological conditions remain elusive. The role of mesenchymal stem cells, growth factors (BMP2, TGF)^14,15^, endoplasmic reticulum stress (ER-stress)^16,17^, elevated serum phosphate (Pi) concentrations triggering osteogenic myoblasts differentiation^18^ and of infiltrating inflammatory cells^19,20^ have been postulated. The dystrophic muscle contains 20 times more macrophages than healthy one^21^. Muscle regeneration requires infiltrating immune cells^22^. But the essential transition from the initial inflammatory to anti-inflammatory immune response is altered in DMD^23,24^, and chronic inflammation contributes to necrosis and fibrosis that exacerbate muscle wasting and impair regeneration^23,25–27^. Interestingly, areas of ectopic calcification in dystrophic mice are localised to myofibers surrounded by macrophages^20^. The tauroursodeoxycholic acid (TUDCA) was shown to attenuate the key alterations implicated in ectopic mineralisation, including inflammatory^28,29^, phosphate-induced fibroblast mineralisation^30^, the ER-stress-induced heart fibrosis^31^ and overload-induced cardiac remodelling^32^. This compound also showed anti-inflammatory effect directly in macrophages^33,34^. Previous studies have reported that TUDCA reduces endoplasmic reticulum (ER) stress and is considered an artificial chaperone preventing abnormal folding of proteins and the unfolded protein response (UPR) development^35^. Given the aberrant calcium homeostasis in dystrophic cells^12,36,37^ combined with ER-stress and UPR responses in the whole muscle of dystrophic mice^38^ we hypothesized that TUDCA administration in the dystrophic mouse model will be protective against ectopic muscle mineralisation or at least will mitigate the ER-stress.

While loss of full-length dystrophin is sufficient to trigger DMD, the dystrophin-null phenotype appears to be exacerbated in both humans and mice^39^ and this includes aggravated ectopic calcification^20,40^. Therefore, we compared TUDCA effects in the dystrophin-null *Dmd*^*mdx-*βgeo^^41^ and *Dmd*^*mdx*^ mice lacking the full-length isoforms only^42^.

While we found substantially elevated level of ER-stress markers in skeletal muscles of both *Dmd*^*mdx-*βgeo^ and *Dmd*^*mdx*^ mice, we unexpectedly observed no impact of TUDCA on these proteins. The levels of osteogenic proteins RUNX2 (Runt-related transcription factor 2), Osterix (transcription factor Sp7) and BMP (bone morphogenic protein) were also found increased in dystrophic muscles, yet TUDCA administration had no effect on these markers either. This lack of effect was not due to an insufficient dose, as TUDCA treatment increased muscle calcification in both dystrophic mouse models. Moreover it had no effect on wild-type counterparts, indicating that the effects are specific for the dystrophic pathology.

## Results

**Fig. 1:**
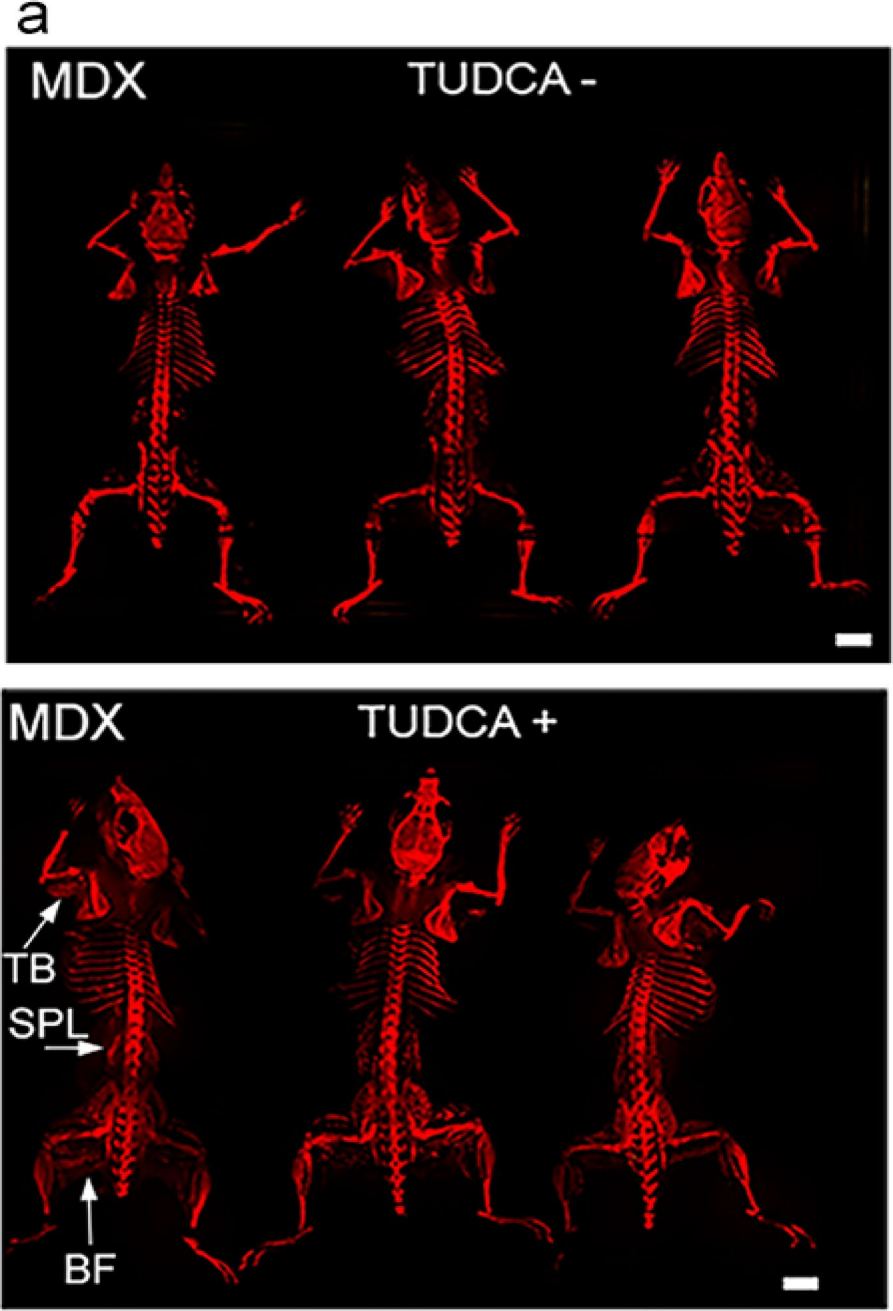

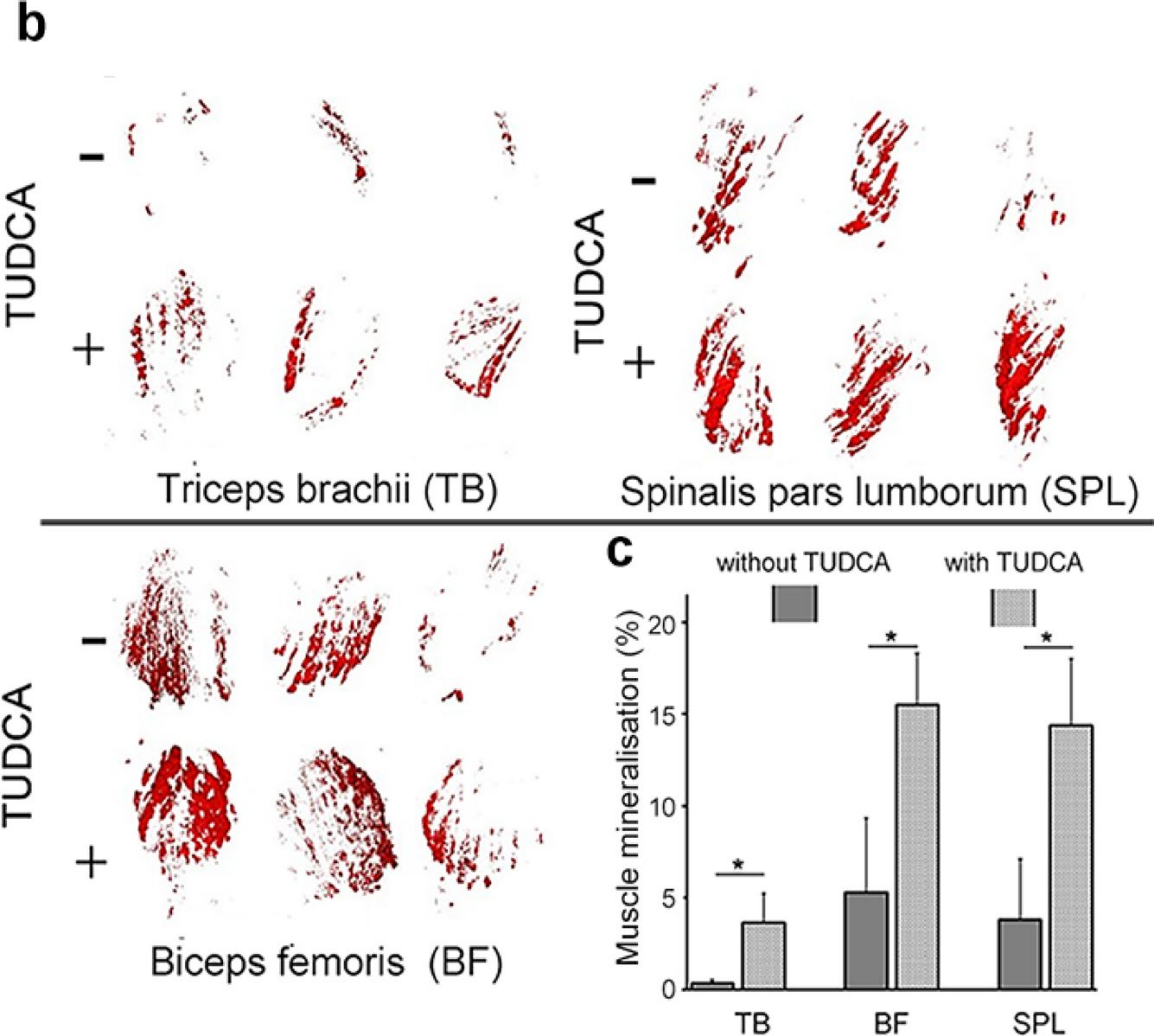
Whole-body analysis of ectopic calcifications in mdx treated with TUDCA. Tissue clearing and Alizarin red staining show distribution of ectopic calcification across the entire musculature. (**a**) Representative epifluorescent images reveal sites of myofiber calcification. (**b**) Three-dimensional light-sheet microscopy data of selected sites of ectopic calcification. (**c**) Percentage mineralization and cumulative frequency distributions in triceps pars brachii, biceps pars femoris and spinalis pars lumborum). Unpaired t-test and two-sample Kolmogorov–Smirnov test were performed. Data are expressed as means ± SD for three animals (independent biological replicates). *P < 0.05.

**Fig. 2:**
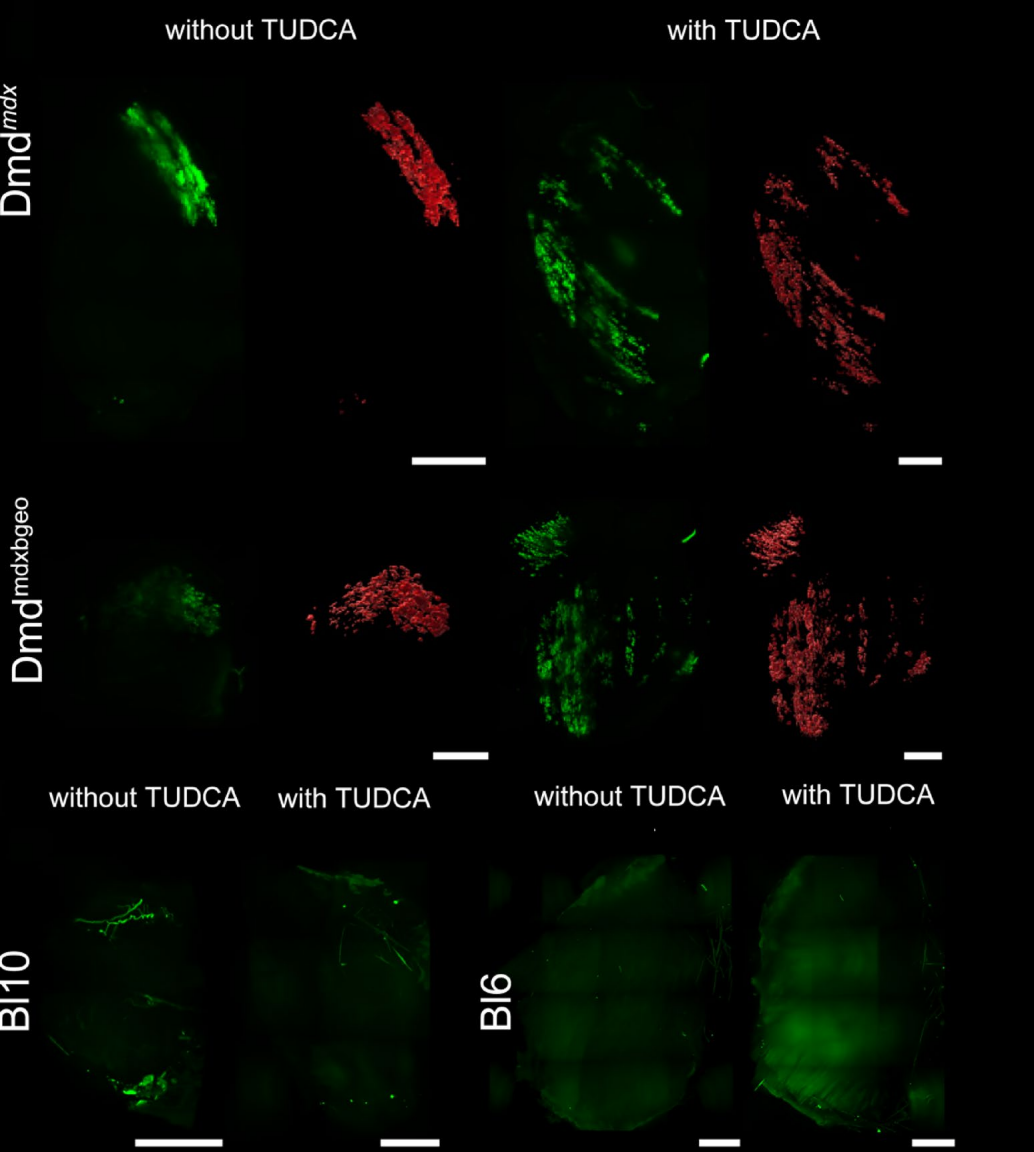
Three-dimensional muscle analysis of ectopic calcifications. Triceps brachii from macroscopically prescreened cleared mice was isolated and imaged in light-sheet fluorescence microscopy for detailed comparison. Column in green presents Alizarin red signal along with autofluorescence of the entire muscle, column in red—segmented Alirazin red signal, n = 6 muscles per group. In the bottom Triceps brachii muscles isolated from w/t animals, both treated and untreated with TUDCA, do not present any calcifications. Scale bar = 1 mm.

TUDCA administration had markedly different effects in dystrophic and wild-type mice. After 4 weeks of TUDCA treatment, there was a significant increase in ectopic calcification in the skeletal muscles throughout the entire *Dmd*^*mdx*^ mouse body (Fig. 1). In contrast, ectopic calcification was absent from dystrophin-positive mice, and TUDCA had no effect on muscle mineralisation in these mice (Figs. 1, 2 the lowest row).

The pro-calcifying effects of TUDCA treatment were also observed in muscles of *Dmd*^*mdx-*βgeo^ dystrophin-null animals (Suppl. 1 and Fig. 2), indicating that this outcome is related to the pathology and the absence of dystrophin. This finding prompted us to investigate the potential dystrophy-specific mechanisms.

Given that macrophages surrounded the areas of ectopic calcification in myofibers in dystrophic mouse muscles^20,40^, we have evaluated the effects of TUDCA in muscle sections stained for mineral deposits using Alizarin Red combined with evaluation of the CD11b marker by Western blot and immunofluorescence. CD11b is used to identify activated macrophages in areas of inflammation^43,44^.

**Fig. 3:**
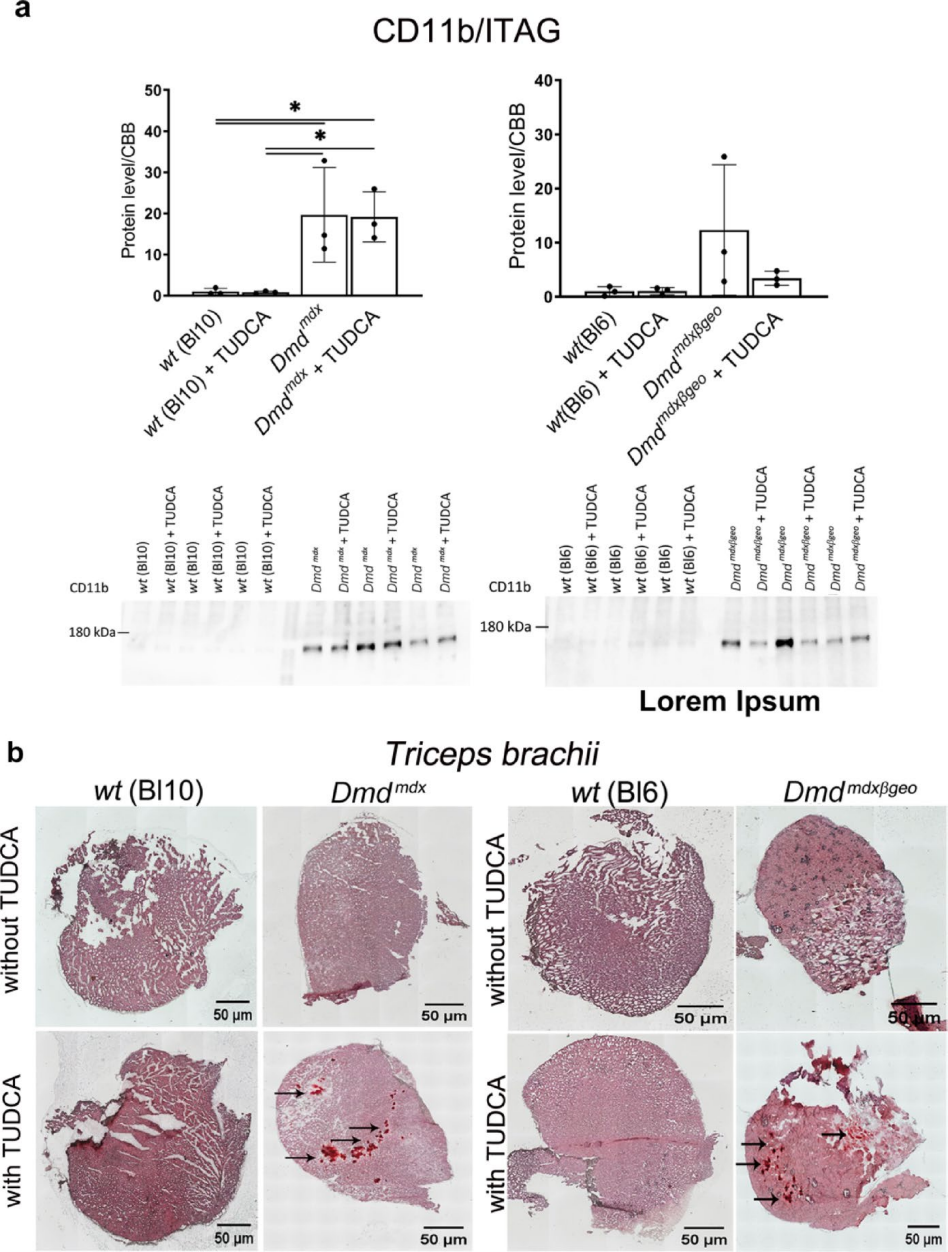

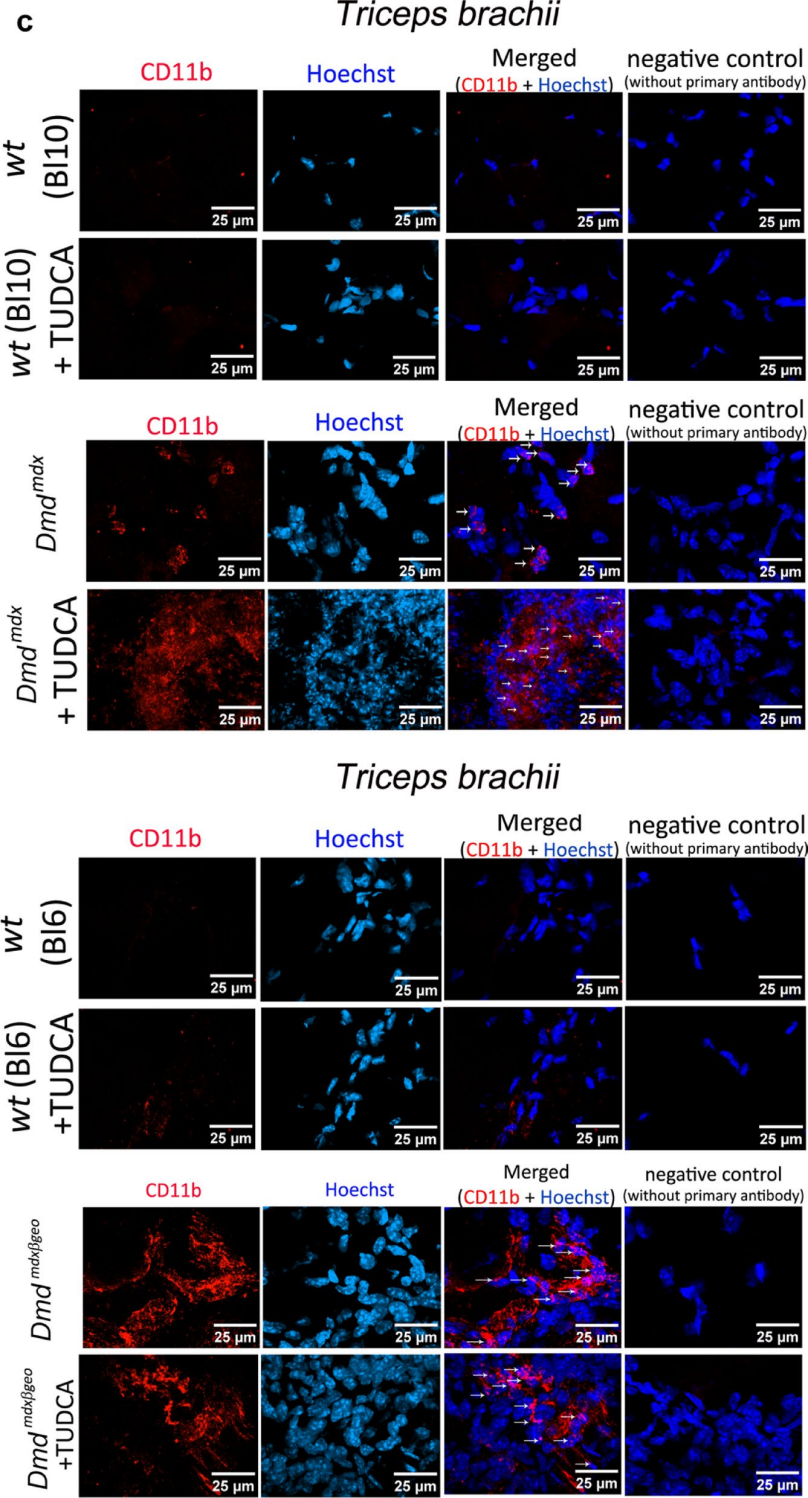
Effects of TUDCA on calcification and inflammation in *Dmd*^*mdx*^ and *Dmd*^*mdx*-βgeo^ triceps muscle. (**a**) Western blot analysis of CD11b protein expression in wild-type and dystrophic triceps. Graphs represent data from three mice and corresponding western blots are shown below. (**b**) Mineralization in C57BL10 and *Dmd*^*mdx*^ (left), BL6 and *Dmd*^*mdx*-βgeo^ (right). Calcium deposits were detected by Alizarin Red S and are visible as red clumps visualized under the light microscope (10 × magnification). (**c**) Confocal images of CD11b immunolocalization. The nuclei were labelled using Hoechst (blue colour). Tissues shown in panels b and c are adjacent sections from the same muscle, which demonstrates the co-existence of mineral deposits and CD11b stained macrophages in the same area.

TUDCA treatment significantly increased the ectopic mineralisation in dystrophic muscles (Fig. 3b). CD11b expression assessed by Western blotting in both dystrophic mice muscles was significantly higher when compared to their wild type counterparts, where this marker expression was largely absent (Fig. 3a). However, TUDCA had no statistically significant impact on CD11b expression levels in either dystrophic or w/t muscles (Fig. 3a, c). This result could reflect the high intra-group variability of this marker in individual muscles analysed in their entirety. CD11b immunofluorescence in muscle sections adjacent to those used for Alizarin Red staining revealed a noticeable difference in areas infiltrated by macrophages. These appeared as foci around the calcium mineral deposits that were present in dystrophic genotypes but absent from wild-type muscles.

Among many biochemical mechanisms behind soft tissue calcification that have been postulated, the ER-stress is particularly prominent^45^. Moreover, there is data showing significant elevation of the ER-stress markers in dystrophic muscles^38^. Therefore, we evaluated the effects of TUDCA treatment on ER-stress markers in *Dmd*^*mdx*^ and *Dmd*^*mdx-*βgeo^ muscles. The wild type mice were also analysed to investigate whether the known effects of TUDCA on the ER-stress are present in skeletal muscle.

**Fig. 4:**
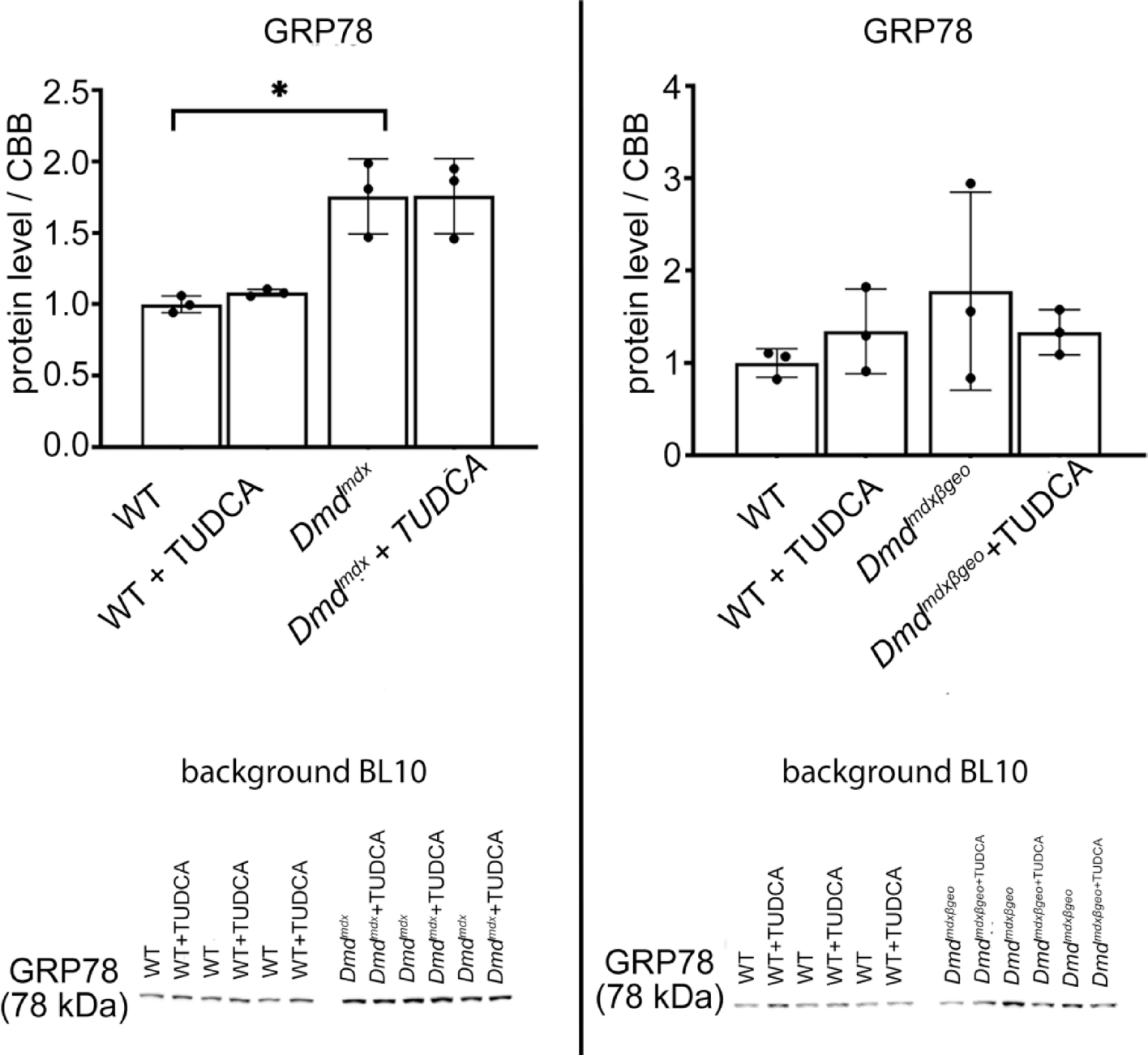
Effect of TUDCA on ER-stress marker levels in lysates from *Dmd*^*mdx*^, *Dmd*^*mdx*-βgeo^ and wild-type mouse triceps muscle. Data show mean values ± S.D. For three independent experiments. Statistical significance at *p* < 0.05 is indicated.

**Fig. 5:**
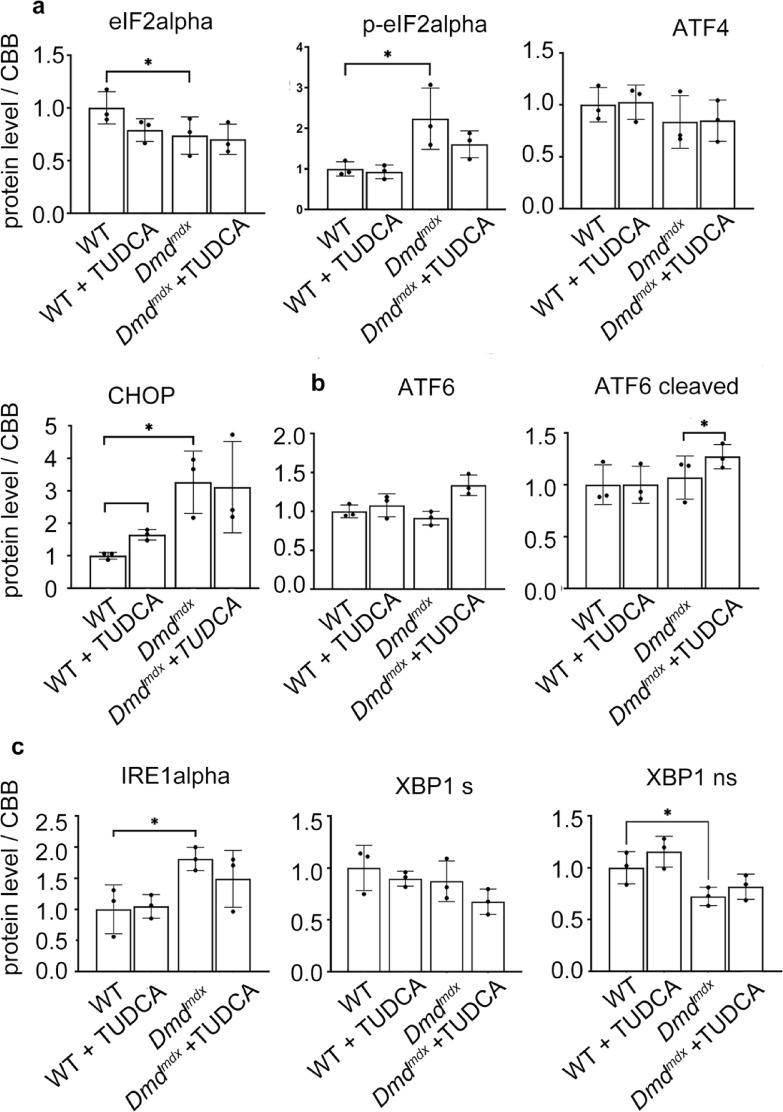
Effect of TUDCA on unfolded protein response marker levels in lysates from *Dmd*^*mdx*^ and wild-type mouse triceps. (**a**, **b**, **c**) Indicate three distinct UPR pathways. Data show mean values ± S.D. for three individual animals. Statistical significance at p < 0.05 is indicated. Corresponding western blots are shown in Suppl. CHOP—C/EBP homologous protein, eIFalpha—Eukaryotic Initiation Factor 2, IRE alpha—inositol-requiring enzyme 1α, XBP1-X-box binding protein 1, ATF4 -Activating transcription factor 4.

**Fig. 6:**
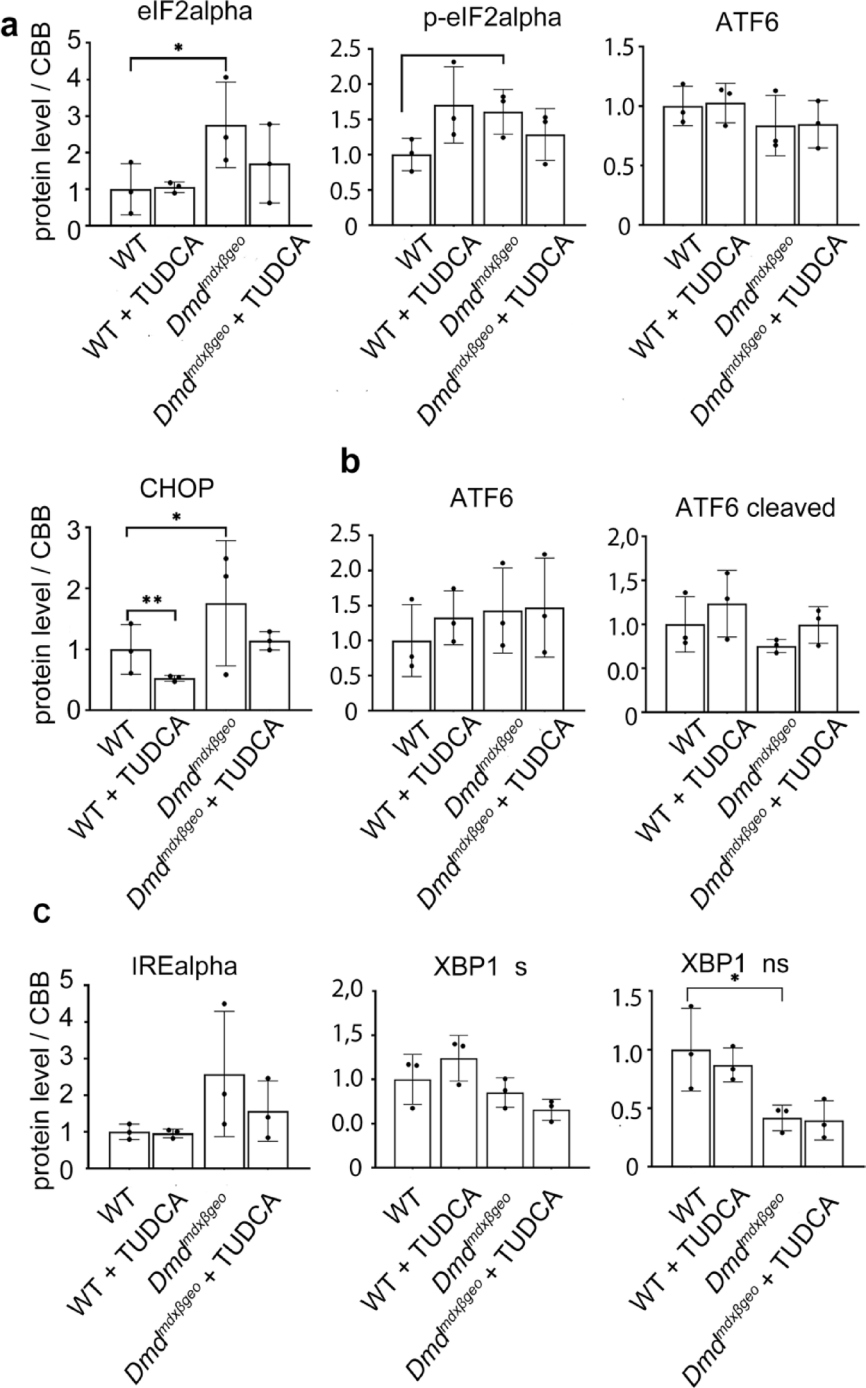
Effect of TUDCA on unfolded protein response marker levels in lysates from *Dmd*^*mdx*-βgeo^ and wild-type mouse triceps. (**a**, **b**, **c**) Indicate three distinct UPR pathways. Data show mean values ± S.D. for three individual animals. Statistical significance at p < 0.05 is indicated. Corresponding western blots are shown in Suppl. 2 and 3. Abbreviations as in Fig. 5.

The expression levels of some proteins from a panel of ER-stress and unfolded protein response (UPR) marker proteins showed significant differences between the dystrophic and wild type muscles. As shown in Fig. 4, GRP78 (Glucose-Regulated Protein 78; ER-stress marker) protein level is substantially increased in *Dmd*^*mdx*^ muscles and less obviously (statistically non-significantly) in *Dmd*^*mdx-*βgeo^. GRP78 is a chaperon, which may initiate unfolded protein response (UPR) under ER-stress conditions. Moreover, cellular levels of proteins belonging to the three UPR pathways are shown for *Dmd*^*mdx*^ and *Dmd*^*mdx-*βgeo^ in Figs. 5, 6, respectively.

Generally, the same set of markers was found altered in both *Dmd*^*mdx*^ and *Dmd*^*mdx-*βgeo^ muscles. However, these differences in expression were not unidirectional, with some markers being up- and other down-regulated.

Crucially, TUDCA treatment had no significant effect on the levels of expression of these ER-stress markers either in dystrophic or wild-type muscles. ATF6 (activating transcription factor 6) showed an increase, but it was statistically significant in *Dmd*^*mdx*^ only. None of the protein levels were significantly altered by TUDCA.

Thus, while the ER-stress may play a role in dystrophic muscle pathology, including ectopic mineralisation, TUDCA does not seem to have any impact on this mechanism.

**Fig. 7:**
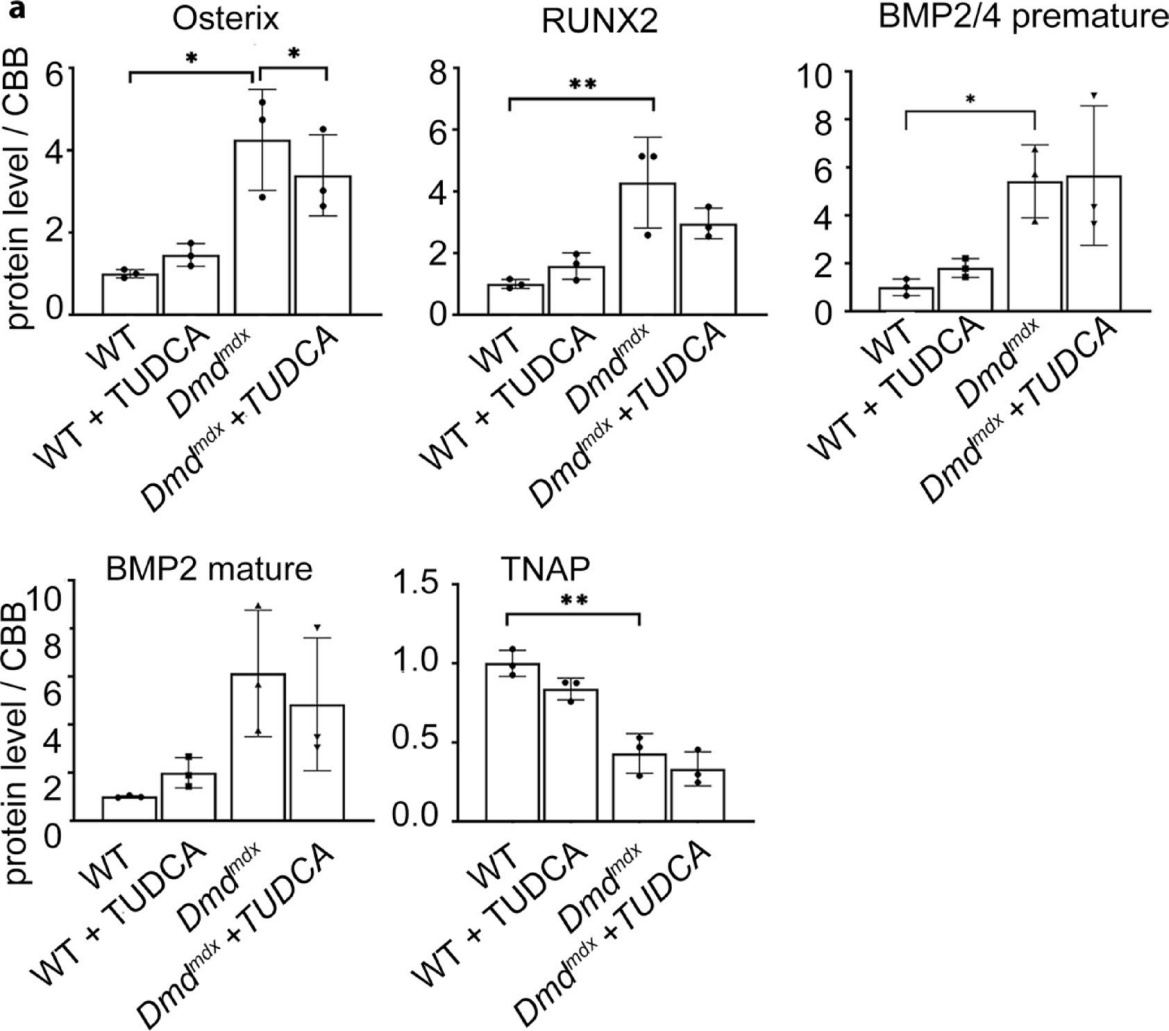

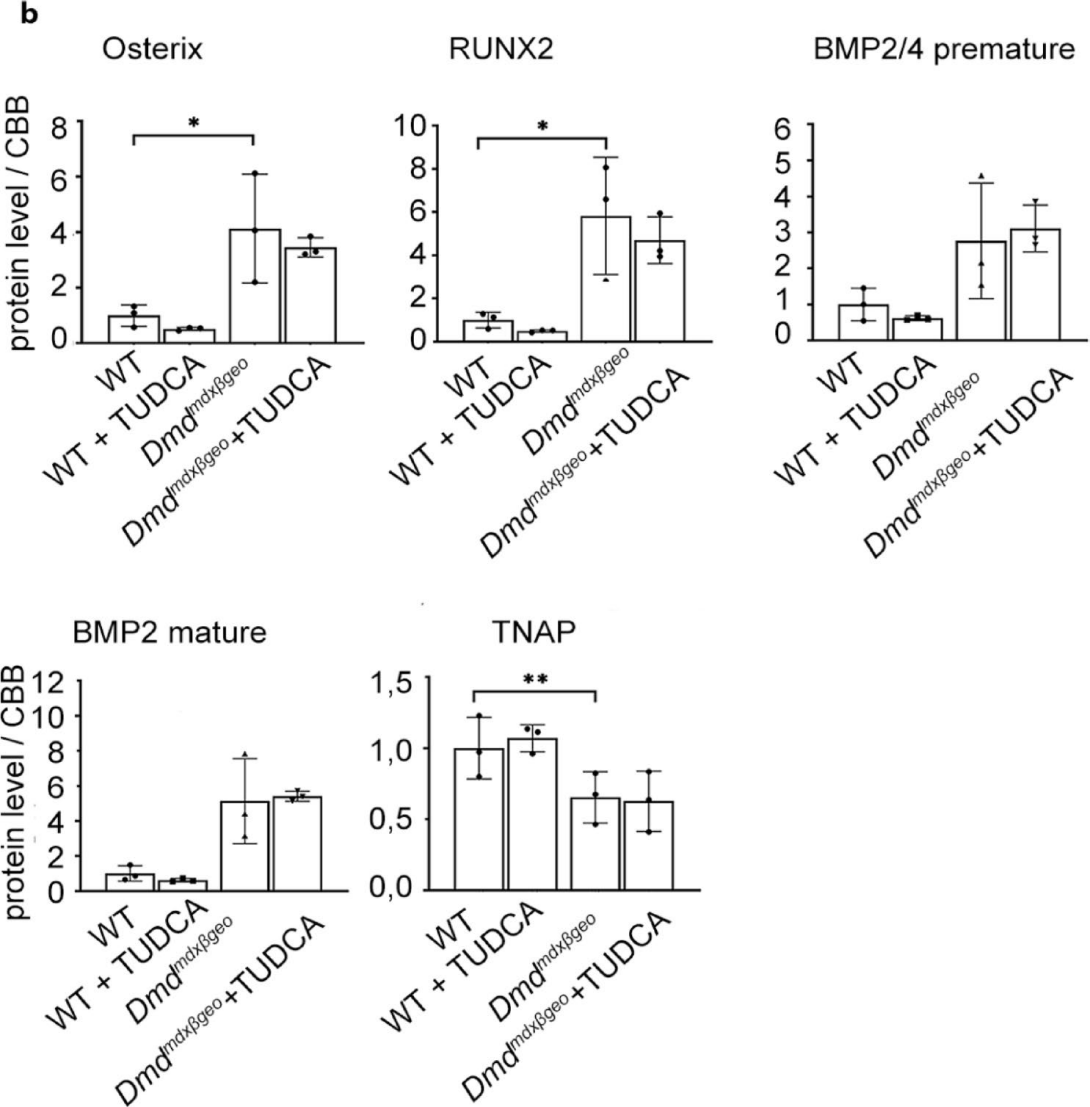
Expression of osteogenic factors Osterix, RUNX2, BMP2 and TNAP in dystrophic muscles. Graphs present relative protein levels in lysates from triceps muscle isolated from w/t and dystrophic mice. Below the relevant Western blots are shown. Upper Row: *Dmd*^*mdx*^ and C57Bl10 samples. Lower row: *Dmd*^*mdx*-βgeo^ and C57Bl6 samples. Graphs show mean values ± S.D. for three independent animals. Statistcal significance is indicated. Corresponding western blots are shown in Suppl. 2 and 3.

Ectopic mineralisation is modified by several local and systemic factors, including elevated levels of specific molecules involved in osteogenic transition of cells^46^. Therefore, we have investigated the expression levels of some key proteins, such as Osterix, RunX2, BMP2 and Tissue-nonspecific alkaline phosphatase (TNAP) in dystrophic muscles, and how these are impacted by TUDCA (Fig. 7).

In both *Dmd*^*mdx*^ and *Dmd*^*mdx-*βgeo^ triceps the levels of Osterix, RunX2 and BMP2 were significantly elevated and TNAP downregulated, when compared to their relevant wild-type counterparts.

**Fig. 8:**
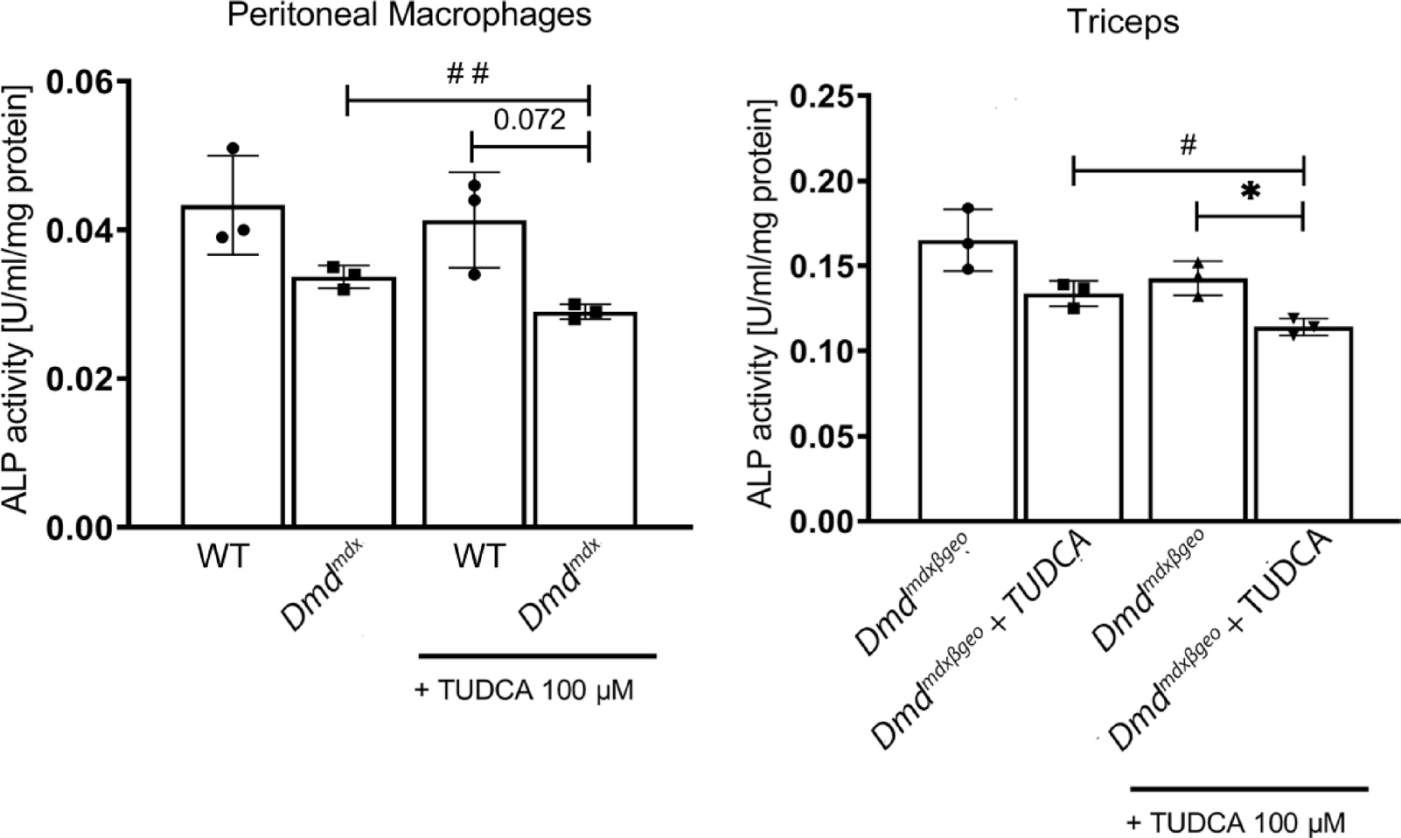
Effect of TUDCA on alkaline phosphatase activity. Enzymatic activity was measured in homogenates prepared from the *Dmd*^*mdx*^ peritoneal macrophages isolated from control (non-treated) mice and *Dmd*^*mdx*-βgeo^ triceps isolated from control or TUDCA-treated mice. To further asses a putative effect of TUDCA on TNAP activity an assay was performed where 100 μM TUDCA was added directly before measurement. Collected data from three individual animals (biological replicates).

As it is shown in Fig. 7 the increased level of osteogenic factors RUNX2, Osterix and BMP2 in dystrophic muscles indicates an increased potential for osteogenesis, which is in line with increased calcification there. This pattern of protein expression was not affected by TUDCA. It is noteworthy that effects of TUDCA on the same marker proteins as shown above were also statistically insignificant in other muscles including *soleus, tibialis anterior and gastrocnemius.* Summarised data concerning the latter one are shown in Suppl. The lower expression of TNAP required further investigation as protein levels may not correspond with the enzymatic activity. Alkaline phosphatase is an enzyme that catalyses pyrophosphate hydrolysis. The changes in the local Pi/PPi ratio could either facilitate or inhibit mineralization, depending on the precise balance of these ions. Pyrophosphate is a powerful inhibitor of hydroxyapatite formation and thus mineralisation. However, the alkaline phosphatase activity was slightly reduced in homogenates prepared from the skeletal muscles of TUDCA-treated *Dmd*^*mdx-*βgeo^ mice. This should result in higher pyrophosphate levels and therefore prevent rather than support ectopic calcification. Thus, this effect of TUDCA contradicts the increased mineralisation in the dystrophic muscles (Fig. 8).

Another potential mechanism of the ectopic mineralisation enhancement by TUDCA could involve an impact of the drug on the inflammatory cells infiltrating the dystrophic muscle. We have shown that macrophages are intricately involved in the ectopic mineralisation process^20,40^. However, also in these cells, TUDCA visibly reduced TNAP activity specifically in dystrophic but not wild type peritoneal macrophages. Thus alkaline phosphatase, either muscle or macrophage derived, seems not to be responsible for TUDCA-evoked muscle mineralisation. Moreover, these findings demonstrate that TUDCA can deregulate calcium homeostasis and increase ectopic mineralisation in a dystrophy-dependent manner.

This led us to the question regarding potential impact of the absence of specific dystrophin isoforms between *Dmd*^*mdx*^ and *Dmd*^*mdx-*βgeo^ treated with TUDCA. The principal difference between these two dystrophic mice is that *Dmd*^*mdx*^ expresses short isoforms, including Dp71 (the shortest protein encoded by the DMD gene) whilst *Dmd*^*mdx-*βgeo^ are dystrophin-null. However, the effects of TUDCA on the expression of key proteins of interest, including indicators of ER-stress and mineralisation, were similar in both dystrophic genotypes, indicating that the pro-calcifying mechanism of TUDCA is independent of the presence or absence of short isoform of dystrophin (see Suppl. 5).

## Discussion

It is likely that ectopic calcification in dystrophic muscles is mediated by a complex interaction of factors, which include the abnormal calcium homeostasis, with dysregulated store-operated calcium entry^37,47^, and also involving muscle, non-muscle and inflammatory cells^36,48–50^.

Another requirement for ectopic calcification is supraphysiological levels of inorganic phosphate, which could be generated by extracellular ATP (eATP) degradation. In the pericellular space, levels of ATP are orders of magnitude greater than in the bulk phase of the surrounding fluid^51,52^ eATP is degraded to inorganic phosphate or pyrophosphate by specific enzymes, and their imbalance due to mutations can lead to ectopic calcification^53^.

However, in dystrophic muscles, this homeostasis is disrupted. Sarcoglycan is a cell membrane ecto-ATPase^54^ and it is lost in DMD. Thus, the already high eATP levels caused by its release from damaged and dying muscles are increased even further, creating dysregulated phosphate metabolism. This local muscle environment prone to highly localised increases in phosphate concentration results in further Pi/PPi imbalance in dystrophic muscles^40,55^.

In this context, the tissue non-specific alkaline phosphatase (TNAP), could have a significant role. This enzyme not only delivers inorganic phosphate released by degradation of a number of phosphorylated substrates but also reduces pyrophosphate concentration. Pyrophosphate is a powerful inhibitor of mineralisation^56^, thus elevation of TNAP activity additionally supports hydroxyapatite formation. However, this mechanism that is highly probable in solid tissues has been recently questioned for serum environment because of very low PPi concentration^57^. Interestingly, we found that TNAP levels were lower in dystrophic muscle and its activity was reduced by TUDCA treatment.

Also, several other factors can modify the mineralization process, including specific pro-osteogenic proteins. We found several of these (RUNX2, Osterix, BMP2 and 4) to be upregulated in dystrophic muscles. In other words, in dystrophic animals, the alkaline phosphatase might be a rate-controlling enzyme, which activity limits minerals formation. In w/t animals, the absent or lower level/activity of other proteins contributing to mineralisation prevent minerals deposition regardless of higher levels of alkaline phosphatase. However, while changes in levels of proteins required for calcification may indicate aberrant regulation of this process in dystrophic mice and explain why dystrophic muscles exhibit some calcification, the TUDCA treatment had no effect on this mechanism.

This finding is in line with pro-calcifying effects of TUDCA, as this compound has a favourable effect on bones and has been tested in the prevention and treatment of osteoporosis^58^ and in a prevention of dystrophy-related osteopenia^59^. In these cases, a stimulatory effect of TUDCA on bone formation was attributed to a specific elevation of TNAP activity by this compound^58^. However, as it is shown in Fig. 8, we did not confirm such a mechanism in dystrophic skeletal muscle. In fact, TUDCA slightly reduced TNAP activity. On the other hand, it cannot be excluded that an ex-vivo assay of TNAP activity does not reflect intramuscular conditions, particularly relative enzyme-TUDCA concentrations and their interaction. Therefore, a stimulatory TNAP-based role of TUDCA, although not confirmed, cannot be completely rejected.

Substantial elevation of dystrophic muscle calcification in animals treated with TUDCA has been convincingly evidenced but this finding stands in opposition to other also well documented anti-calcifying effects of this compound in soft tissues. Available data indicate that the ectopic mineralisation of soft tissues is attributed to the endothelial reticulum stress. Indeed, increased content of ER-stress markers was previously described in D2-mdx mice which exhibit particular tendency for muscle mineralisation^38^. In mdx muscle proportion between different ER-stress markers was substantially deregulated and, against the expectations^30^, this dysregulation was not prevented in animals treated with TUDCA.

These results, although do not exclude a role of ER-stress itself in dystrophic muscle calcification, they bring into question a putative effect of TUDCA on the ER-stress-related dystrophic-muscle mineralisation. In addition, lack of preventive action of TUDCA on ER-stress in dystrophic mice stands in opposition to the described effects of this compound^35,60–62^. It seems to be worth consideration that TUDCA-induced reduction of the ER-stress in other studies was observed upon acute insult such as radiation^63^ tunicamycin^61^ or thapsigargin treatment^64^. In the case of dystrophic animals, they have adapted to the dystrophin deficiency through many generations, an elevated level of ER-stress markers may reflect the established *status quo*, which in fact is “normal” in these mice and adjusted to a broadly modified cell metabolism. Therefore, the term “ER-stress” may not be adequate for describing such a stable state of cells with mutation in dystrophin gene. Thus, TUDCA as an additional chaperone has no effect on this well-established steady-state.

It must also be stressed that in experiments shown here TUDCA was administered orally, thus it could undergo metabolic transformations in the liver. This was suggested before by Kars et al., who tested effect of TUDCA on insulin resistance in muscle cells and did not observed any changes in cellular level of ER-stress markers although muscle sensitivity to insulin was substantially improved^65^. These observations differ from those when TUDCA was administered intraperitoneally, or it was added to the cell media. However, even if TUDCA reduces ER stress, as suggested by others, it still would have not explained an elevation of mineral deposits in TUDCA-treated mdx mice.

The degree of dystrophic muscle calcification seems to be slightly higher in DMD *mdx*^*β-geo*^ than in DMD *mdx* mice but effects of TUDCA on all parameters tested were very similar in both strains of the mice. It suggests that TUDCA acts independently of Dp71 protein expressed in *mdx* but not DMD *mdx*^*β-geo*^ mouse muscles.

Western blot analysis revealed substantial and TUDCA-independent increase of CD11b protein levels in lysates of mdx triceps compared to the equivalent muscle from dystrophin-positive mice. However, microscopic analysis of muscle slices revealed more calcium mineral deposits in TUDCA-treated animals, which colocalised with increased CD11b specific signal. This observation needs further evaluation but it may suggest a role of inflammatory cells in TUDCA-induced mineralisation. It has been supported by the differential effects of TUDCA on TNAP activity in wild-type and dystrophic macrophages (Fig. 8). It seems to be particularly important as it was found that macrophages infiltrating dystrophic muscles protect against rather than support tissue mineralisation, and this protective effect is strongly enhanced upon a treatment of cells with P2RX7 agonist even when muscle P2RX7 was selectively ablated^40^.   On the other hand, mineralisation in differentiating, conditionally immortalised dystrophic mice myoblasts grown in high-phosphate medium was observed in the absence of macrophages. Because these cells have elevated level of P2RX7 a putative role of this receptor in the myoblasts-dependent mineralisation as well as a potential effect of TUDCA in these cells require additional study.

To conclude, skeletal muscles of dystrophic mice undergo spontaneous calcification. Given the elevated levels of proteins supporting calcification and modified pattern of ER-stress markers in dystrophic muscles, this ectopic calcification may be explained by the changes in expression of ER-stress related proteins and proteins involved in calcium mineral accumulation. These mechanisms are, however, not affected or, as for TNAP, are reduced by TUDCA. Nevertheless, treatment in dystrophic muscles but not muscles of dystrophin-positive animals significantly increased calcification. Notably, since TUDCA is used as a dietary supplement, and has been tested as a treatment in various clinical settings^66,67^, our results have therapeutic implications, as TUDCA may trigger or exacerbate ectopic calcification in muscles damaged in mechanisms different than dystrophy. Further work investigating the molecular mechanism of TUDCA is underway.

## Methods

### Animals and TUDCA treatment

All methods were carried out in accordance with relevant guidelines and regulations as described by the ARRIVE guidelines (PLoS Bio 8(6), e1000412, 2010). They also complied with the Polish Law on Experiments on Animals, which implements the European Council Directive, and were approved by the 1^st^ Local Ethical Committee for Animal Experimentation in Warsaw (Permit Number: 1279/2022). (Permit Number: 1279/2022).

Four-week-old male dystrophic mice strains C57BL/10ScSn-Dmd/J (mdx) and C57BL/6-DmdGt (ROSAbgeo)1Mpd/J (*Dmd* ^*mdx-*βgeo^) and the appropriate dystrophin-positive (w/t) animal strains C57BL/10ScSnJ and C57BL/6ScSnJ, respectively (Jackson Laboratory, Jacksonville, USA) were used. Randomly selected group of mice received in their diet tauroursodeoxycholic acid (TUDCA) dissolved in drinking water at 2 mg/ml. Fresh TUDCA solution was administered every other day for 4 weeks^31^. Remaining animals obtained pure water to drink. Total amount of TUDCA per one mouse varied around 250 mg. After 4 weeks of such a treatment the 8-week-old animals were killed by cervical dislocation preceded by deep anaesthesia with isoflurane. Selected muscles (Triceps branchii and Gastrocnemius) were prepared respectively from the forelimbs and hindlimbs of dystrophic and w/t animals.

### Whole-body tissue clearing, imaging and analysis

Clearing procedure was performed as described previously^68^. Briefly, animals were deeply anesthetized with i.p. injection of lethal dose of sodium pentobarbital (100 mg/kg), subjected to cardiac perfusion, and subjected to fixation with 4% PFA followed by 3 days of clearing with CUBIC reagent-1A^69^ and 1 day of 0.03% (w/v) AR (Alizarin Red S) staining dissolved in fresh clearing solution. Finally, specimens were placed for 3 days of gentle shaking with fresh clearing solution at 37 °C in an incubator to remove the excess of unbound AR. Noteworthy, AR stains calcium deposits and does not detect subcellular calcium accumulation, which may occur due to muscle inactivity, particularly in dystrophic muscles. Respective muscles were manually dissected using scalpel. Next, images were collected with customized light sheet apparatus^70^ and LaVision Ultramicroscope II with CUBIC-R1A as a imaging solution and analyzed using Imaris (Bitplane) “surface” function with following parameters—surface grain size = 3.00 µm, enable eliminate background = true, diameter of largest sphere = 100 µm.

### Tissue non-specific alkaline phosphatase (TNAP) activity assay

TNAP activity was measured according to the protocol attached to the Alkaline Phosphatase Assay Kit (Colorimetric) ab 83369 from Abcam. Briefly, the piece of muscle was homogenized in ALP Assay Buffer. Then, the samples were centrifuged and the supernatant was used to measure TNAP activity according to the protocol precisely described by the company. The TNAP activity in muscle was measured spectrophotometrically using p-nitrophenyl phosphate (PNPP) as a substrate. The absorbance was measured in a 96-plate reader (OD 405 nm) after 1 h incubation in RT in the dark. The TNAP activity was normalized with protein concentration and expressed as U/ml/mg protein, where U = nmol p-nitrophenyl (PNP)/min.

### Western blotting

Whole muscles were homogenised in the lysis buffer (1 × LysisM, 1 × protease inhibitor cocktail, 2 × phosphatase inhibitor cocktail (all Roche), 2 mM sodium orthovanadate (Sigma), then suspending with the use of automatic pipets followed by repeatedly forcing through the syringe needle and incubation of the suspension on ice for 20 min. After centrifugation (15,000 × g, for 20 min at 4 °C) protein concentration in collected supernatants was determined using a Bradford protein assay (Bio-Rad). Remaining supernatants were mixed with sample buffer at 3:1 v /v ratio, heated for 5 min at 95 °C and chilled on ice and stored at − 80 °C. Proteins (25 μg of each sample) were separated on 0.1% SDS polyacrylamide gels (6–12% w/v depending on the molecular mass of protein) and electroblotted onto Immobilon-PVDF Transfer Membrane (Merck Millipore). Blots were blocked in 5% w/v non-fat milk or 5% BSA (Albumin, Bovine Serum, 12659, Merck Millipore) powder solved in 1 × TBST, 0.01% v /v Tween-20 (Sigma) for 1 h at room temperature (RT) prior to probing with appropriate primary antibody diluted in 2.5% milk or 5% BSA depends on antibodies (incubated at 4 °C overnight with agitation). To identify proteins of interest following primary antibodies were applied: osterix (OSX diluted 1:250, sc-393325), RUNX2 (1:250, sc-3903551), BMP-2/4 (1:500, sc-137087), TNAP (1:1000, sc-166261), IRE1alpha (1:250, sc-390960), eIF2alpha (1:1000, sc-133132) from Santa Cruz, GRP78 (1:50 000 dilution, ab21685 Abcam), ATF4 (1:1000, 108-35-1-AP Proteintech), ATF6 (1:500, NBP1-75478), XBP1 (1:5000, NBP1-77681) from Novus Biologicals, p-eIF2alpha (1:1000, 119A11 Cell signalling), cd11b/ITGAM (1:500, A1581 ABclonal). Then membranes were washed (3 ×) with 1 × TBST for 10 min each wash and incubated with anti-Rabbit (diluted 1:5000, ab6721) or anti-Mouse (1:3000, ab6728) from Abcam horseradish peroxidase-conjugated secondary antibody for 1 h at RT. Specific protein bands were visualized using luminol-based substrates (Millipore) and images obtained using a Fusion FX (Vilber Lourmat). Intensity of particular bands was normalised using Commassie staining as described by Welinder and Ekblad^71^. Densitometric analyses of specific protein bands were made using exposure times within the linear range and the integrated density measurement function of BIO-1D (Vilber Lourmat).

### Fluorescent microscopy

Frozen muscles were cut into 10 µm slices on polysine adhesion microscope slides. After rinsing twice with PBS (w/o calcium and magnesium), the slices were fixed in a 4% w/v paraformaldehyde solution (PFA) in PBS for 15 min at room temperature (RT). Then permeabilized using PBS with 0.1% Triton X-100 for 30 min and blocked in a 5% v /v goat serum (GS, Normal Goat Serum, S-1000, Vector Laboratories IVD) in PBS for 1 h at RT. Then incubated overnight at 4 °C with primary antibodies cd11b/ITGAM (A1581 ABclonal, 1:100 diluted in blocking buffer,). The secondary antibody (diluted 1:1000 in 5% GS, Alexa Fluor®594 goat anti-Mouse, Thermo Fisher Scientific) was added for 1 h RT in the dark. The slides with slices of muscles were rinsed for 15 min 3 times under agitation between each step of the IHC protocol. The nuclei were labelled with Hoechst 33342 (Thermo Fisher cat. no. H1399). After staining, slices were covered by coverslips in Glycergel Mounting Medium (H-1000 VectaShield®, Vector Laboratories) before imaging. Images were obtained using a confocal microscope (Zeiss Spinning Disk Confocal Microscope) and image analysis was performed using Image J software (1.54p version). Fluorescence filters were used as follows: Alexa Fluor 594 ex 561 nm laser and em. at 575–625 nm. Hoechst. ex 405 nm laser and em. 432–482 nm.

### Statistical analysis

Data are expressed as a mean value ± standard deviation (SD). Statistical significance was assessed by Student’s t-test, a *p*-value of < 0.05 was considered statistically significant; n = 3, where “n” represents the number of repeated experiments on muscles derived from three different mice. Where indicated reported p values were calculated using two-way ANOVA with Tukey post hoc test. Values were FDR (False Discovery Rate factor) adjusted for multiple comparisons.

## Supplementary Information

Below is the link to the electronic supplementary material.

Supplementary Material 1

## Data Availability

All data generated or analysed during this study are included in this published article and its supplementary files. Because of their size, the entire western blot membranes are available on request from the corresponding author.
