# Supplementary material for: Tauroursodeoxycholic acid-induced increase in ectopic muscle mineralization occurs exclusively in dystrophic muscles and is independent of endoplasmic reticulum stress
Source: Sci Rep. 2025 Oct 28;15:37704. doi: 10.1038/s41598-025-21534-0 (PMC12569086; doi:10.1038/s41598-025-21534-0)
Supplement: Supplementary file 1 — Supplementary Material 1 [file 41598_2025_21534_MOESM1_ESM.pdf]

Supplement

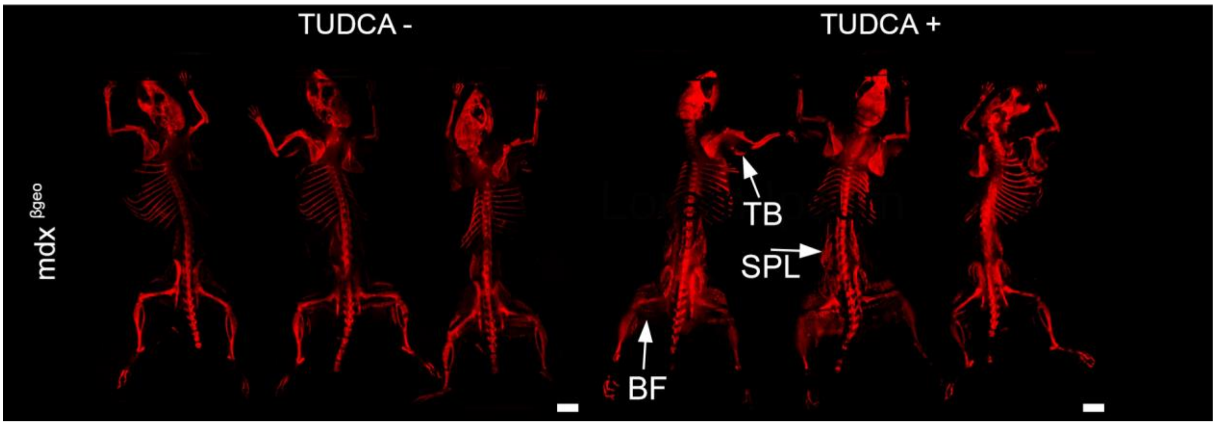

***Suppl. 1. Whole-body visualisation of ectopic calcifications in Dmd^{mdxbetageo} mice treated with TUDCA**.*

TB – Triceps branchii, SPL - Spinalis pars lumborum, BF – Biceps femoris

Scale bar = 1 mm.

Supplement for Fig. 5

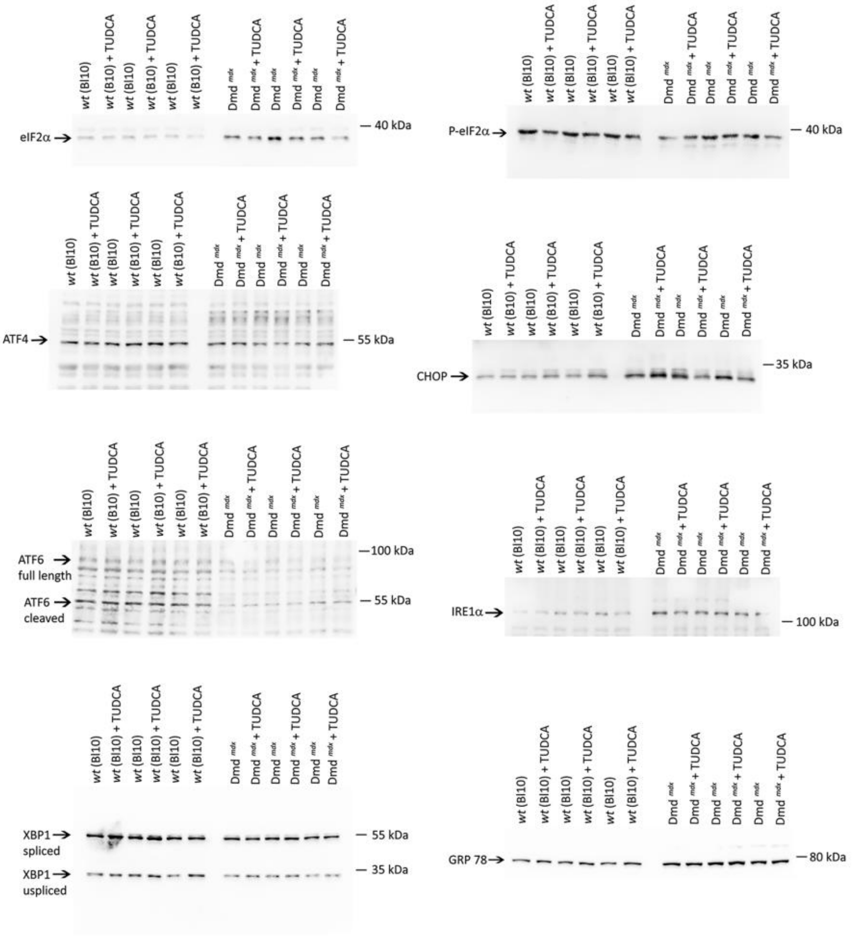

Supplement for Fig. 6

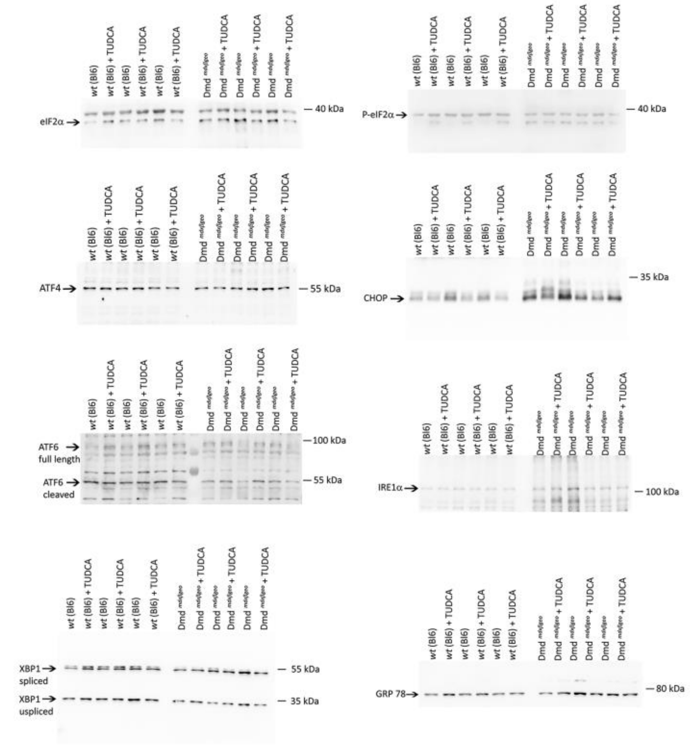

Supplement for Fig. 7 a and b

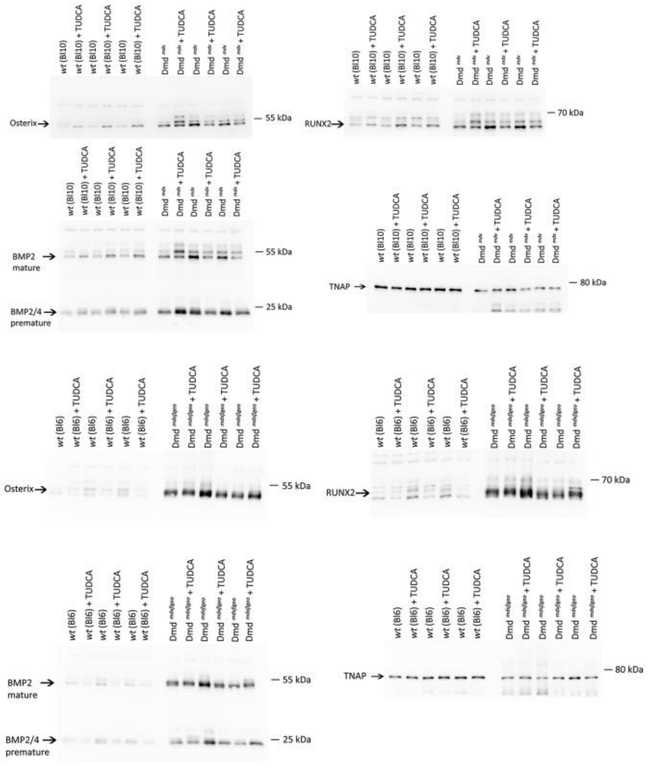

Suppl. 2. Western blots for Figures 5, 6 and 7.
All strips cover the whole width of the membrane. Protein mass markers are non-visible because of chemiluminescent detection. All markers are visible in white light imaging and also after CBB staining (see below).

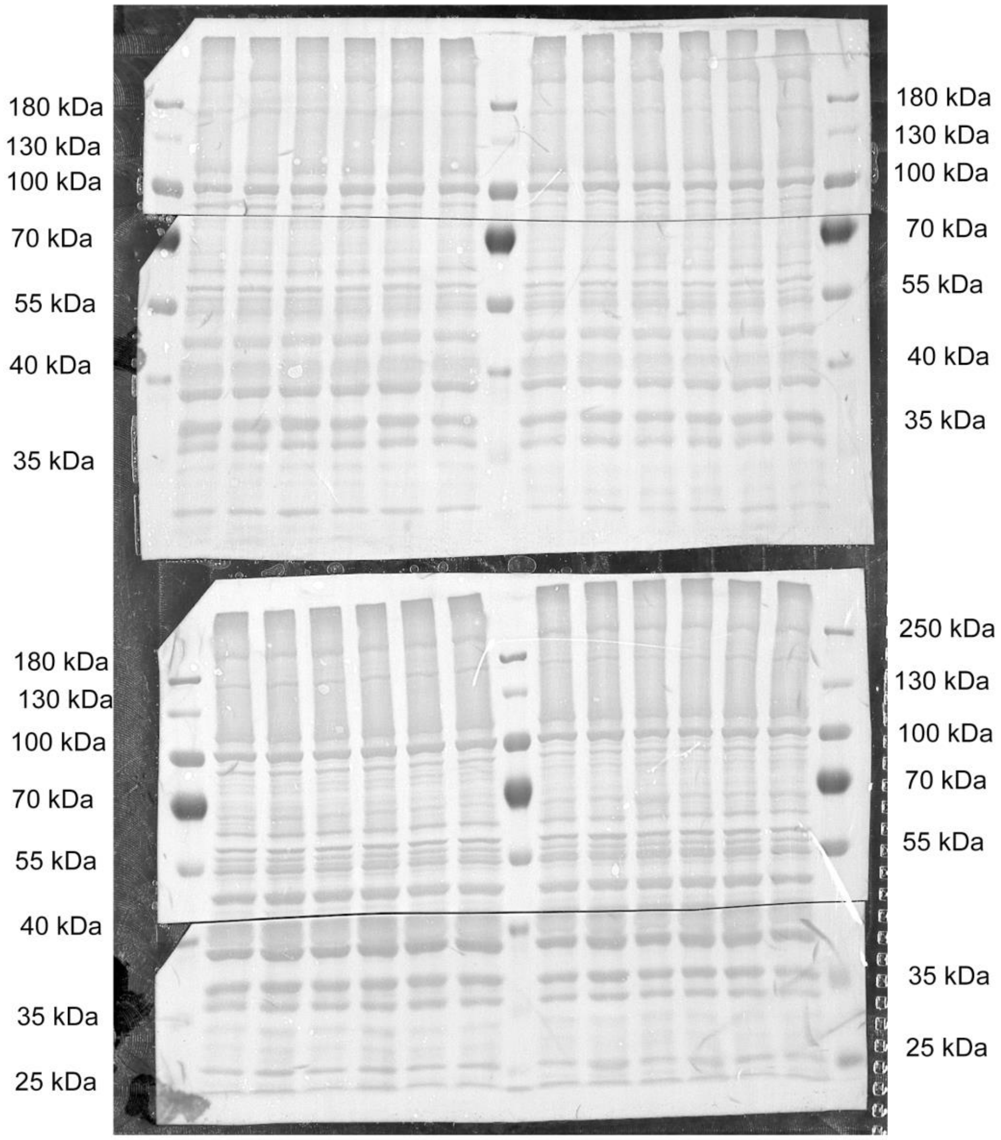

Suppl. 3. Two independent representative blots stained with Coomassie Brilliant Blue.

In a case of each protein the first western blot was made with the use of intact membrane, to confirm proper localisation of the bands.
In next analyses blots were cut prior to hybridisation with antibodies. Then all strips were put together and stained with CBB to visualise whole membrane. Such a procedure was performed for all experiments we made.

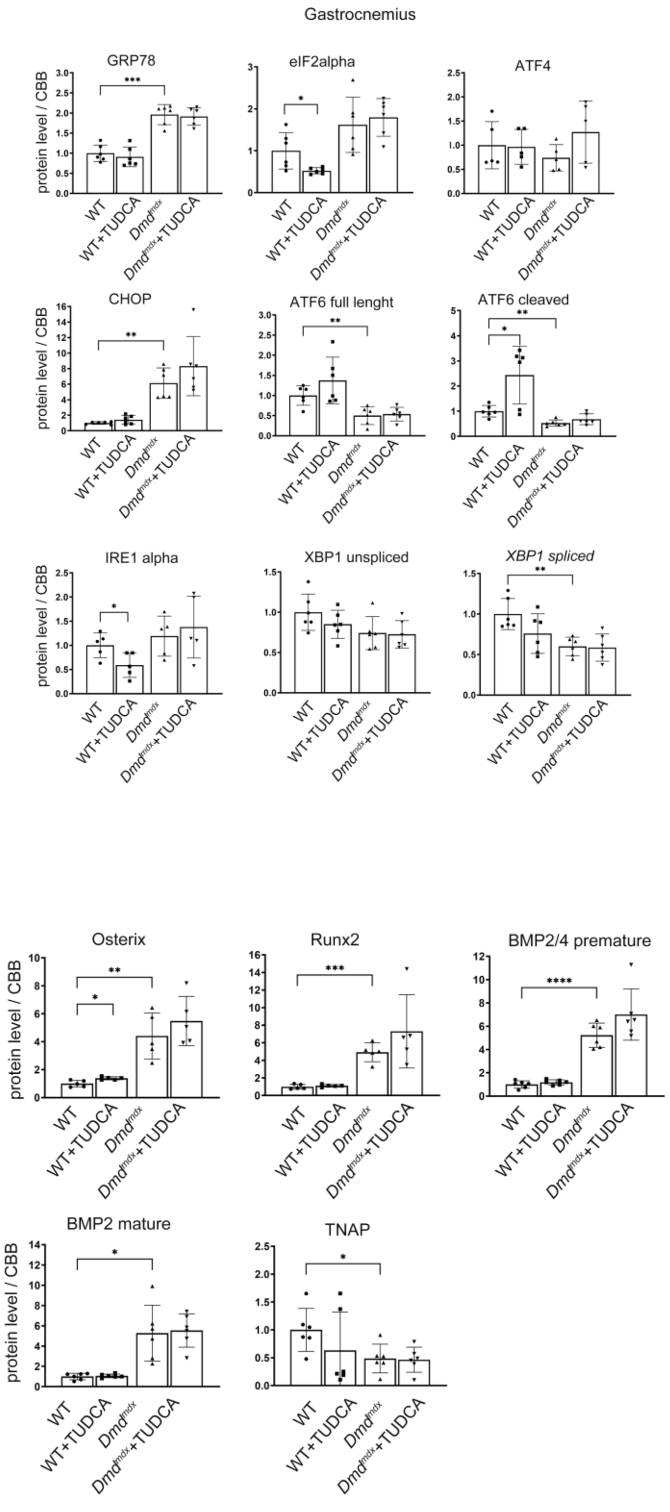

Suppl. 4. Effect of TUDCA on unfolded protein response and osteogenic marker levels in lysates from Dmd^{mdx} and wild-type mouse gastrocnemius.

Graphs present relative protein levels in lysates from gastrocnemius muscle

Similarly as it was shown for the triceps muscle ER-stress marker levels in mdx gastrocnemius are substantially affected in comparison to dystrophin-positive (w/t) muscle. Although the pattern of these changes is different in both type of muscles, TUDCA does not influence

dystrophic muscle at least in this matter. Therefore the enhanced calcification of them in TUDCA treated mice cannot be explained as an effect of this compound on the ER stress. Moreover, TUDCA was expected to reduce ER-stress and therefore prevent muscle calcification instead of its magnification.

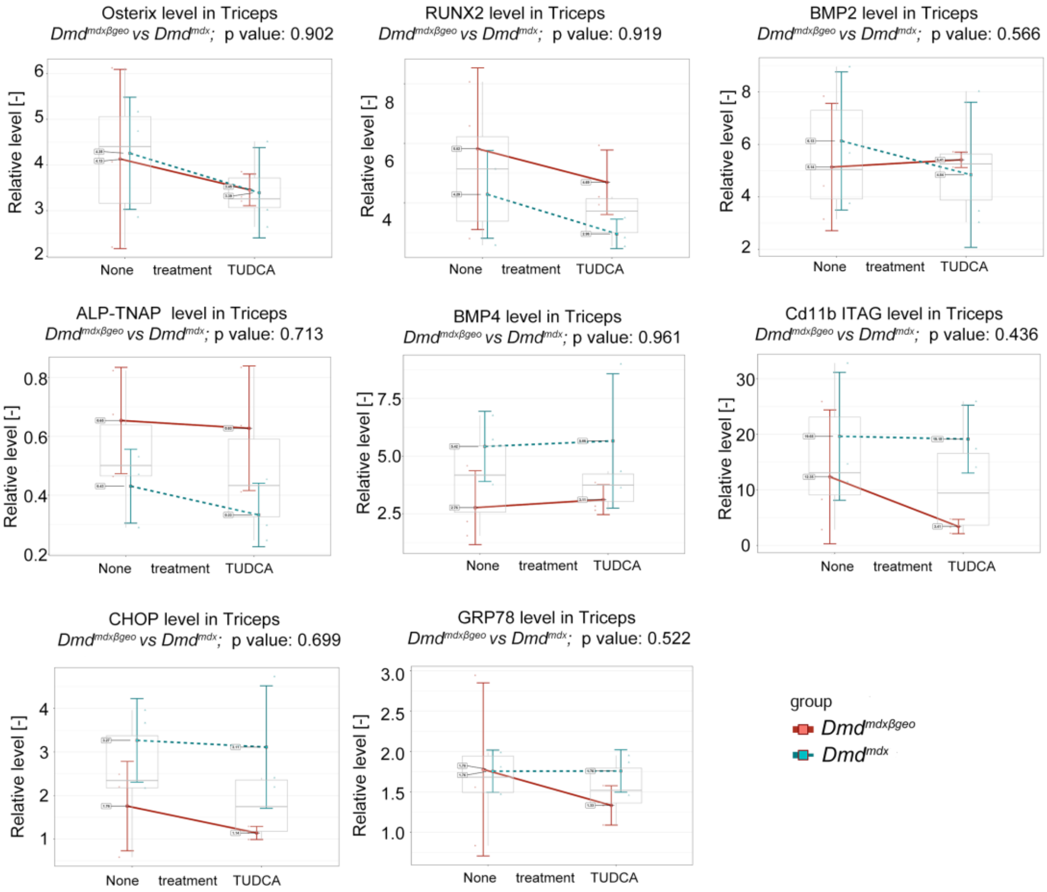

Suppl. 5. Difference in response to TUDCA treatment between Dmd^{mdx} and Dmd$^{mdx\beta geo}$ mouse as a normalized protein level to C57Bl10 and C57Bl6, respectively.

Reported p values were calculated using two-way ANOVA with Tukey post hoc test. Values were FDR adjusted for multiple comparisons.
